# Apremilast: A Phosphodiesterase 4 Inhibitor for the Treatment of Psoriatic Arthritis

**DOI:** 10.1007/s40744-014-0005-4

**Published:** 2014-12-09

**Authors:** Philip J. Mease

**Affiliations:** Swedish Medical Center and University of Washington School of Medicine, 601 Broadway, Suite 600, Seattle, WA 98122 USA

**Keywords:** Apremilast, Otezla, PALACE clinical trial program, Phosphodiesterase 4 inhibitor, Psoriatic arthritis

## Abstract

**Introduction:**

Psoriatic arthritis (PsA) is a spondyloarthritis that occurs in up to 30% of psoriasis patients. Patients with PsA are at risk for decreased quality of life due to both joint and skin symptoms, impaired physical function and disease progression. Treatments include non-steroidal anti-inflammatory drugs, conventional systemic disease-modifying anti-rheumatic drugs (DMARDs) such as methotrexate, and biologic agents, including tumor necrosis factor-α inhibitors. The most recently introduced treatment option is apremilast, an oral phosphodiesterase 4 inhibitor.

**Methods:**

This review provides an in-depth discussion of apremilast’s mechanism of action, and evidence of its clinical efficacy and safety from the Psoriatic Arthritis Long-term Assessment of Clinical Efficacy (PALACE) phase III pivotal clinical trials (PALACE 1, 2, and 3).

**Results:**

These trials demonstrate that apremilast is effective for the treatment of active PsA, despite prior conventional DMARDs or biologic treatment. The primary efficacy end point, a 20% improvement from baseline in modified American College of Rheumatology response criteria at Week 16, was achieved by significantly greater proportions of patients treated with apremilast 20 mg twice daily (BID) and apremilast 30 mg BID versus placebo in PALACE 1, 2, and 3. Improvements in this and other clinical and patient-reported end points, including swollen and tender joint counts, Psoriasis Area and Severity Index score, physical function, and quality of life, were maintained, extending over 52 weeks of treatment among patients initially randomized to apremilast. Apremilast’s safety profile has been acceptable, with diarrhea and nausea being the most common adverse events, with no evidence for an increased risk of infection or need for laboratory monitoring. The PALACE pivotal data indicate that apremilast presents a new option for the treatment of PsA that may be appropriate for use early in the treatment ladder. Ongoing PALACE open-label extension trials of up to 4 years will characterize the long-term clinical effects and safety of apremilast therapy.

**Funding:**

Celgene Corporation, Summit, NJ, USA.

**Electronic supplementary material:**

The online version of this article (doi:10.1007/s40744-014-0005-4) contains supplementary material, which is available to authorized users.

## Introduction

Psoriatic arthritis (PsA) is an inflammatory spondyloarthritis associated with psoriasis. Estimates of PsA prevalence among patients with psoriasis vary widely (6.6–48.0%) [1, 2]. In one study that included rheumatologist assessment, PsA was found in 30% of a sample of patients with psoriasis [3]. Many individuals with PsA are undiagnosed [3–5]. In the Prevalence of Psoriatic Arthritis in Adults with Psoriasis: An Estimate from Dermatology Practice (PREPARE) study, 41% of the detected PsA cases had not been diagnosed before study entry [3].

PsA is a multi-domain disease, affecting the musculoskeletal axis, skin, and nails [6–8]. Symptoms of PsA may include joint pain and swelling, enthesitis, dactylitis and, in those patients with active skin involvement, itching, all of which may lead to impaired physical function, work limitations, emotional distress and social embarrassment. These disease manifestations impact patients’ health-related quality of life (HRQOL) to a similar extent as other arthropathies and chronic diseases such as diabetes or heart disease [9–11]. To achieve a more timely diagnosis of PsA, primary care physicians and dermatologists may benefit from education and simple questionnaires that help identify signs of inflammatory arthritides, flag signs and symptoms of PsA such as enthesitis and dactylitis, and identify immune-modulated joint inflammation [12].

Currently, a range of systemic therapies are available for the treatment of PsA, including conventional oral therapies and biologic agents. Long-term use of these agents may be limited by safety and tolerability issues, variable efficacy, route of administration (injection/infusion with biologic agents) and cost [13–15]. Methotrexate is among the most widely used conventional oral therapies [16]. However, evidence of the effectiveness of methotrexate for treatment of PsA is limited and conflicting [17–21], and methotrexate has been associated with risks of hepatic, pulmonary and bone marrow toxicity [16]. Biologic agents that act as tumor necrosis factor (TNF) inhibitors—etanercept, adalimumab, infliximab, golimumab and certolizumab—have demonstrated efficacy in improving the signs and symptoms of PsA in large, well-controlled clinical studies [12, 14, 22], but patients sometimes experience waning efficacy over time [13] or rare but serious adverse events (AEs), such as infection and non-melanoma skin cancer [14]. Other factors that limit the use of biologic agents include injection-related anxiety and cost [15].

Rheumatologists, dermatologists and patients acknowledge a need for new medications that show efficacy, can be tolerated, and are relatively safe. The clinical successes and unmet needs observed with conventional oral therapies and biologic agents have directed research and therapeutic development, resulting in a search for new agents that target components of the pathophysiologic pathways of PsA with fewer of the limitations characterizing current treatments.

## Apremilast

Apremilast (Otezla^®^, Celgene Corporation, Summit, NJ, USA) is an orally available phosphodiesterase 4 (PDE4) inhibitor approved by the US Food and Drug Administration (FDA) in March 2014 for the treatment of active PsA in adults, and in September 2014 for the treatment of moderate to severe plaque psoriasis in patients who are candidates for phototherapy or systemic therapy [23]. This review will focus on the efficacy and safety of apremilast in the treatment of PsA.

**Fig. 1:**
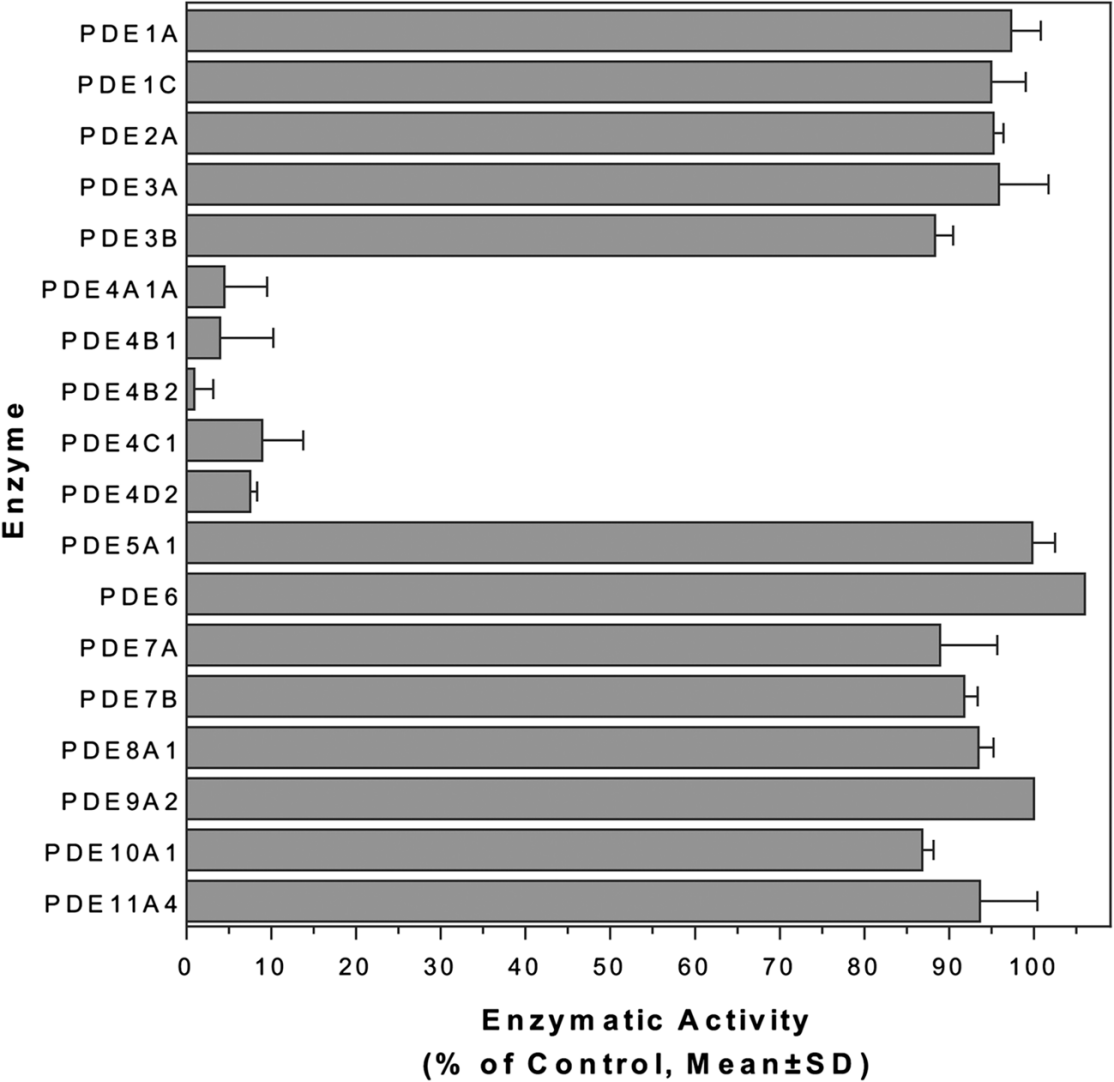
Effect of apremilast on phosphodiesterase (PDE) enzymatic activity. Enzymatic reactions were carried out in 100 nM cyclic adenosine monophosphate as a substrate, except for PDE5A1 and PDE9A2 (100 nM cyclic guanosine monophosphate) and retinal rod PDE6 (100 μM). Data are shown as the mean and standard deviation (SD) from assays performed in duplicate. Reproduced with permission from Schafer et al. [24]

The only known mode of action of apremilast is selective PDE4 inhibition [24], mediated via PDE4 binding at apremilast’s dialkoxyphenyl pharmacophore, a feature shared with other PDE4 inhibitors [24]. PDE enzymatic activity is the sole means of cyclic adenosine monophosphate (cAMP) degradation to AMP in cells [25]. cAMP is an intracellular secondary messenger responsible for a wide array of cellular functions, including regulation of inflammatory signaling and immune homeostasis [24]. PDE4 comprises a group of isoforms among many in the PDE enzyme family. In vitro studies demonstrate that apremilast selectively inhibits activity of several PDE4 isoforms, including the long (4B1, 4C1), short (4B2, 4D2) and super-short (4A1A) isoforms, with no significant inhibition of other PDEs, cell surface receptors or kinases (Fig. 1) [24].

**Fig. 2:**
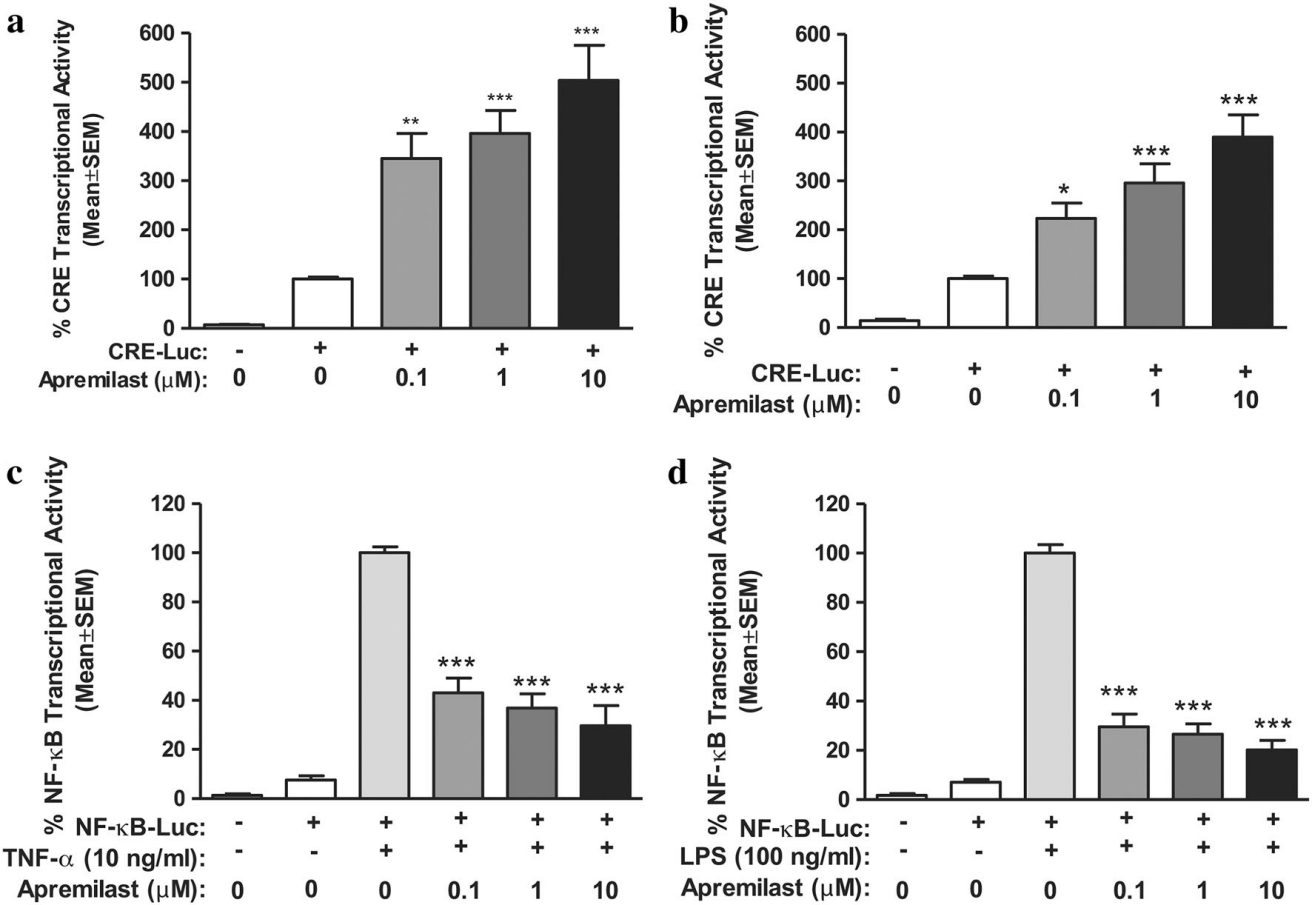
Effect of apremilast on cyclic adenosine monophosphate response element-binding protein and nuclear factor-kappa B (NF-κB) transcriptional activity in **a** Jurkat T cells and **b** T helper 1 (THP-1) monocytic cells at 6 h. All treatment groups were compared with dimethyl sulfoxide by one-way analysis of variance (ANOVA) followed by Dunnett’s multiple comparison post-test (*n* = 4); *p < 0.05; **p < 0.01, ***p < 0.001. Effect of apremilast on NF-κB–driven transcriptional activity in **c** Jurkat T cells and **d** THP-1 monocytic cells at 6 h. All treatment groups were compared with tumor necrosis factor (TNF)-α **c** or lipopolysaccharide (LPS) **d** by one-way ANOVA followed by Dunnett’s multiple comparison post-test (*n* = 4); ***p < 0.001 versus TNF-α or LPS. *CRE* cyclic adenosine monophosphate response element, *SEM* standard error of the mean. Reproduced with permission from Schafer et al. [24]

Each PDE4 isoform is found, in relatively varying degrees, in immune cells such as monocytes, T cells and neutrophils [25]. In human peripheral blood mononuclear cells, apremilast-mediated PDE4 inhibition, in response to prostaglandin E_2_, results in elevated intracellular cAMP concentrations [24]. In turn, the apremilast-mediated rise in cAMP concentrations causes downstream changes in gene expression via induction of cAMP response element-binding/activating transcription factor 1 phosphorylation and binding to the cAMP response element DNA sequence, thus driving cAMP response element-dependent gene expression and inhibition of nuclear factor-κB transcriptional activity (Fig. 2) [24].

**Fig. 3:**
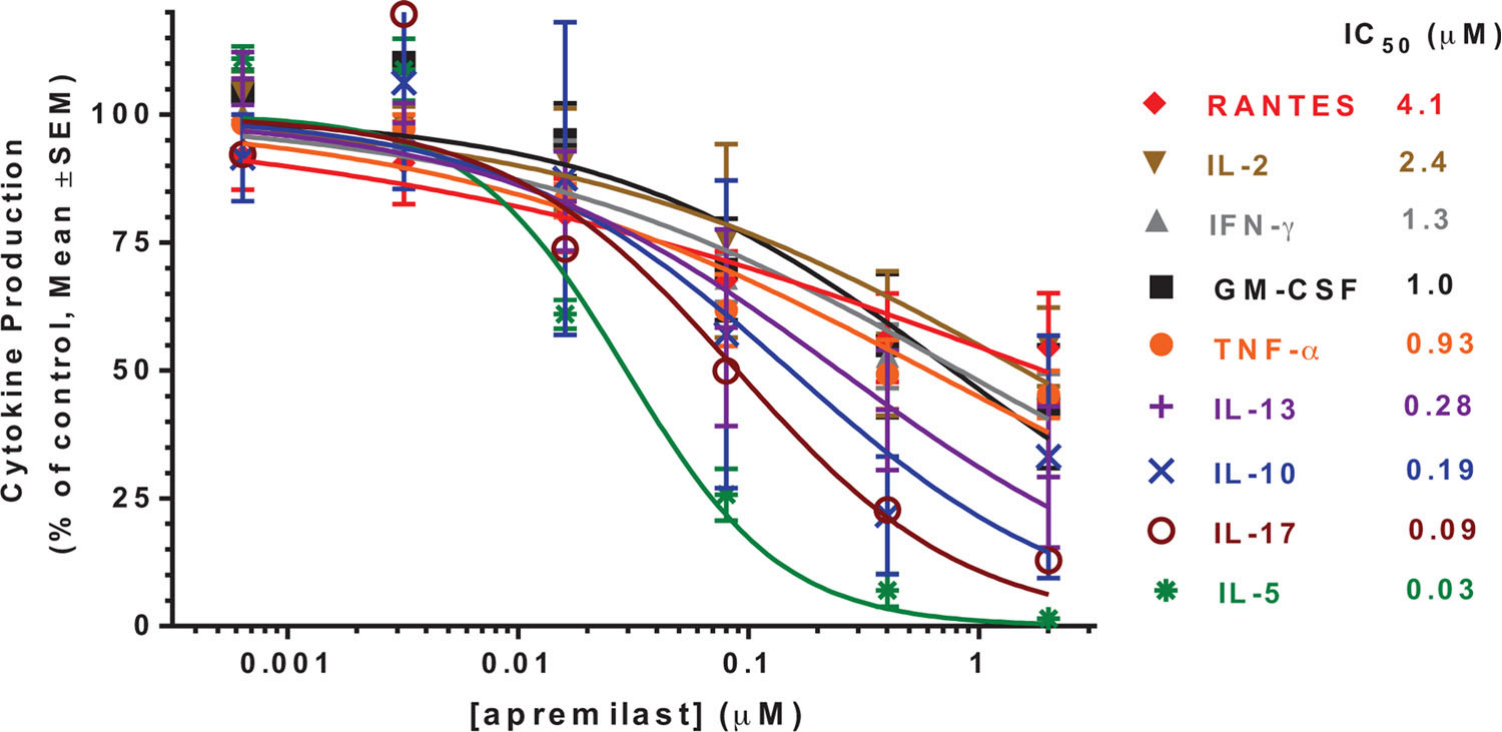
Apremilast inhibition of Th1, Th2, and Th17 cytokines from primary human T cells stimulated via anti-CD3 antibody. Results were averaged using data from four separate T-cell donors. *GM-CSF* granulocyte macrophage colony-stimulating factor, *IC*_*50*_ half maximal inhibitory concentration, *IFN*-*γ* interferon gamma, *IL* interleukin, *RANTES* regulated on activation, normal T cell expressed and secreted, *SEM* standard error of the mean, *TNF* tumor necrosis factor. Reproduced with permission from Schafer et al. [24]

Specific changes in protein production observed in human peripheral blood monocytes with apremilast include inhibition of lipopolysaccharide-stimulated production of TNF-α and cytosine phosphodiester-guanine (CpG) oligonucleotide-stimulated production of interferon-α [24]. Apremilast did not significantly inhibit immunoglobulin G or immunoglobulin M production in normal B-cell cultures [24]. In vitro experiments using CD3-stimulated T cells demonstrate that apremilast inhibits T-cell-derived cytokines, including interleukin (IL)-2, IL-5, IL-13, and IL-17, as well as granulocyte macrophage colony-stimulating factor (GM-CSF) and interferon-γ (Fig. 3) [24], while expression of anti-inflammatory mediators IL-10 and IL-6 is increased with apremilast [26]. Despite its broad inhibition of inflammatory cytokine production, other in vitro experiments show that apremilast has no effect on T-cell or B-cell clonal expansion or on antibody responses in vivo using the antigen-specific mouse B-cell transfer model [24], suggesting that key aspects of adaptive immune system responses may be relatively unaffected by apremilast treatment.

In the psoriasis and arthritis in vivo models, apremilast administration leads to reductions in epidermal thickening, proliferation and histologic psoriasiform features [26], and blocks synovial inflammation, cartilage damage and bone erosion [27]. Overall, the broad nature of apremilast-mediated changes to gene transcription and protein production act to intracellularly regulate numerous inflammatory mediators associated with psoriatic disease [24].

## Clinical Efficacy of Apremilast in Psoriatic Arthritis: Phase III Clinical Trials

**Fig. 4:**
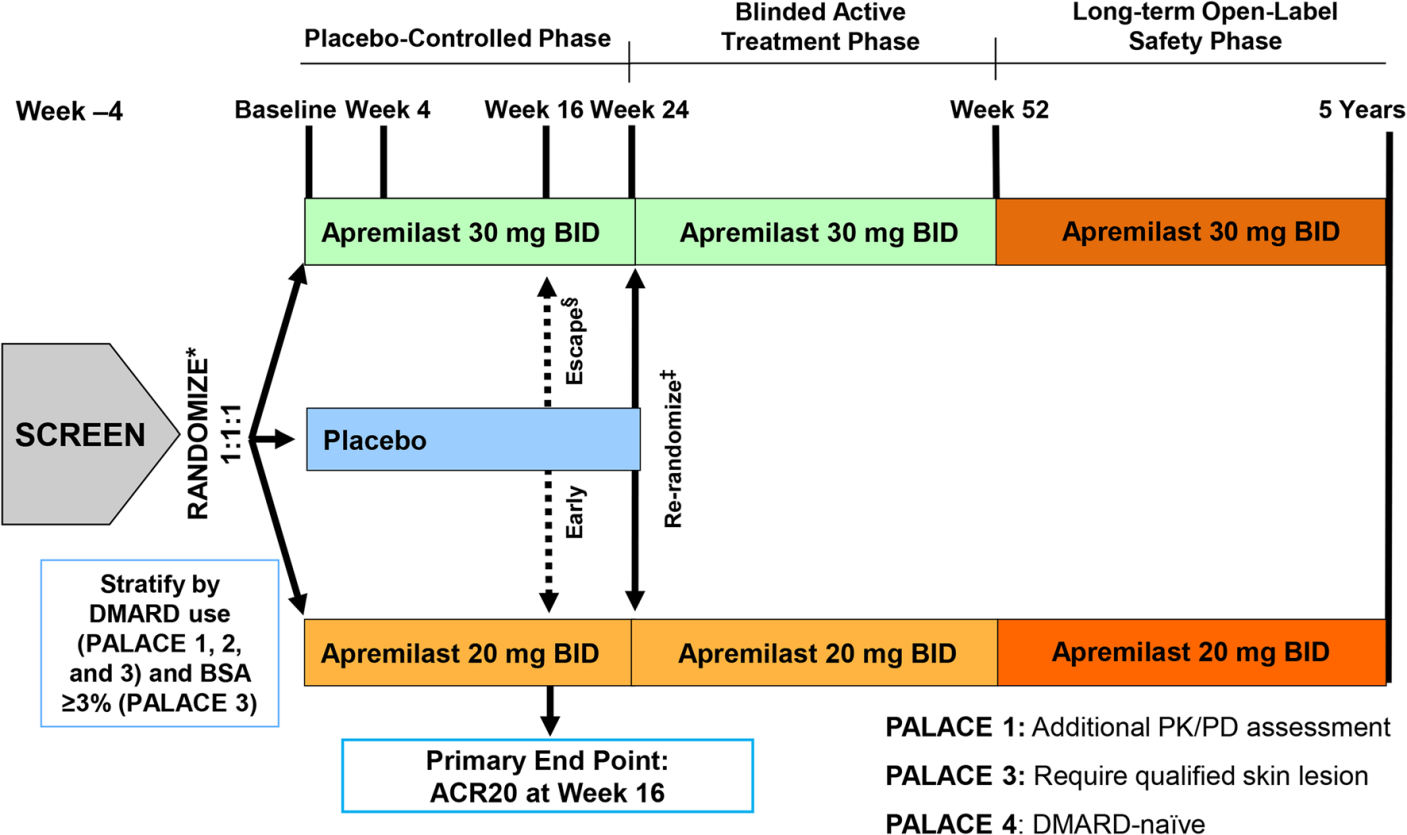
Overview of the Psoriatic Arthritis Long-term Assessment of Clinical Efficacy (PALACE) trial design. ^*^All doses were titrated over the first week of treatment. ^§^Placebo patients whose swollen and tender joint counts had not improved by ≥20% were considered non-responders at week 16 and were required to be re-randomized (1:1) to apremilast 20 mg twice daily (BID) or 30 mg BID if they were initially randomized to placebo. Apremilast-treated patients remained on their initial dose. ^‡^At week 24, all remaining placebo patients were re-randomized to apremilast 20 mg BID or 30 mg BID. *ACR20* American College of Rheumatology 20, *BID* twice daily, *BSA* body surface area, *DMARD* disease-modifying anti-rheumatic drug, *PD* pharmacodynamics, *PK* pharmacokinetic. Reproduced with permission from Gladman et al. [28], Edwards et al. [29]

The effectiveness of apremilast in the treatment of active PsA in adults has been evaluated in the Psoriatic Arthritis Long-term Assessment of Clinical Efficacy (PALACE) phase III clinical trial program. The PALACE program comprises 4 similarly designed, placebo-controlled trials (Fig. 4) [28, 29]. PALACE 1, 2, and 3 are pivotal trials that enrolled patients with active PsA despite prior conventional disease-modifying anti-rheumatic drugs (DMARDs) and/or biologic agents, including a percentage of biologic efficacy failures (Table 1) [28, 30]; PALACE 4 enrolled DMARD-naïve patients [29]. Patients were excluded from the PALACE trials if they had presence of: (1) erythrodermic, guttate, or generalized pustular psoriasis, or rheumatic disease other than PsA; (2) a history of other clinically significant disease or presence of other major uncontrolled disease; (3) active tuberculosis, history of incompletely treated tuberculosis, or significant infection within 4 weeks of screening (no screening for latent tuberculosis was required); (4) malignancy (except treated basal cell or squamous cell skin carcinoma or early forms of cervical carcinoma with no recurrence in 5 years); (5) alanine aminotransferase and/or aspartate aminotransferase >1.5 times the upper limit of normal (ULN) and total bilirubin >ULN or albumin <lower limit of normal; and (6) prior therapeutic failure of >3 agents for PsA or >1 TNF blocker [31]. The PALACE 4 trial will be discussed in a separate publication. This review article will focus on the three pivotal PALACE trials.

**Table 1:** PALACE patient demographic and disease characteristics [30, 33, 51, 52]

	Placebo			Apremilast 20 mg BID			Apremilast 30 mg BID			
	PALACE 1 *n* = 168	PALACE 2 *n* = 159	PALACE 3 *n* = 169	PALACE 1 *n* = 168	PALACE 2 *n* = 163	PALACE 3 *n* = 169	PALACE 1 *n* = 168	PALACE 2 *n* = 162	PALACE 3 *n* = 167
Age, mean (years)	51.1	51.2	49.5	48.7	50.9	49.6	51.4	50.5	49.9
Female (%)	47.6	53.5	53.8	49.4	58.3	53.3	54.8	58.6	52.7
BMI, mean, kg/m^2^	31.1	29.5	29.5	30.9	29.3	30.1	30.6	29.2	29.2
Duration mean, years										
Psoriatic arthritis	7.3	7.8	6.8	7.2	7.8	7.7	8.1	6.8	7.5
Psoriasis	15.7	17.8	17.8	15.5	17.9	18.3	16.5	18.7	17.1
Swollen joint count (0–76), mean	12.8	9.2	11.1	12.5	10.4	11.4	12.8	10.3	11.6
Tender joint count (0–78), mean	23.3	18.0	18.3	22.2	20.3	20.8	23.1	21.8	20.9
HAQ-DI (0–3), mean	1.2	1.2	1.2	1.2	1.1	1.1	1.2	1.2	1.2
PASI score (0–72), mean	9.1	8.6	7.6	7.4	7.4	7.6	9.2	7.8	7.9
Baseline DMARD use (%)	65.5	71.1	59.8	66.1	69.9	61.5	63.1	69.8	60.5
Prior use of DMARDs (biologic-naïve) (%)	71.4	84.9	71.6	76.8	82.8	69.8	73.8	82.7	74.3
Prior use of biologics (%)	24.4	14.5	28.4	22.0	17.2	29.6	24.4	14.2	25.7
Prior biologic failures (%)	11.3	5.0	7.1	8.3	6.1	10.7	8.3	4.3	8.4

The *n* reflects the number of randomized patients who received ≥1 dose of study medication; actual number of patients available for each parameter may vary

*BID* twice daily, *BMI* body mass index, *DMARD* disease-modifying anti-rheumatic drug, *HAQ-DI* Health Assessment Questionnaire-Disability Index, *PALACE* Psoriatic Arthritis Long-term Assessment of Clinical Efficacy, *PASI* Psoriasis Area and Severity Index

In PALACE 1, a total of 504 patients were randomized and received one or more doses of placebo, apremilast 20 mg twice daily (BID), or apremilast 30 mg BID, comprising the intent-to-treat (ITT) population [30]. As shown in Fig. 4, patients whose swollen and tender joint counts had not improved by ≥20% at Week 16 were considered non-responders and were required to be re-randomized (1:1) to apremilast 20 mg BID or 30 mg BID if they were initially randomized to placebo, or continued on their initial apremilast dose. At Week 24, all remaining placebo patients were re-randomized to apremilast 20 mg BID or 30 mg BID; all patients received blinded treatment through to Week 52 [30]. Upon completion of the 52-week, double-blind period, patients were eligible to enter a long-term follow-up phase for a total treatment duration of up to 5 years. Concomitant use of stable doses of conventional DMARDs was permitted throughout the trial [30].

**Fig. 5:**
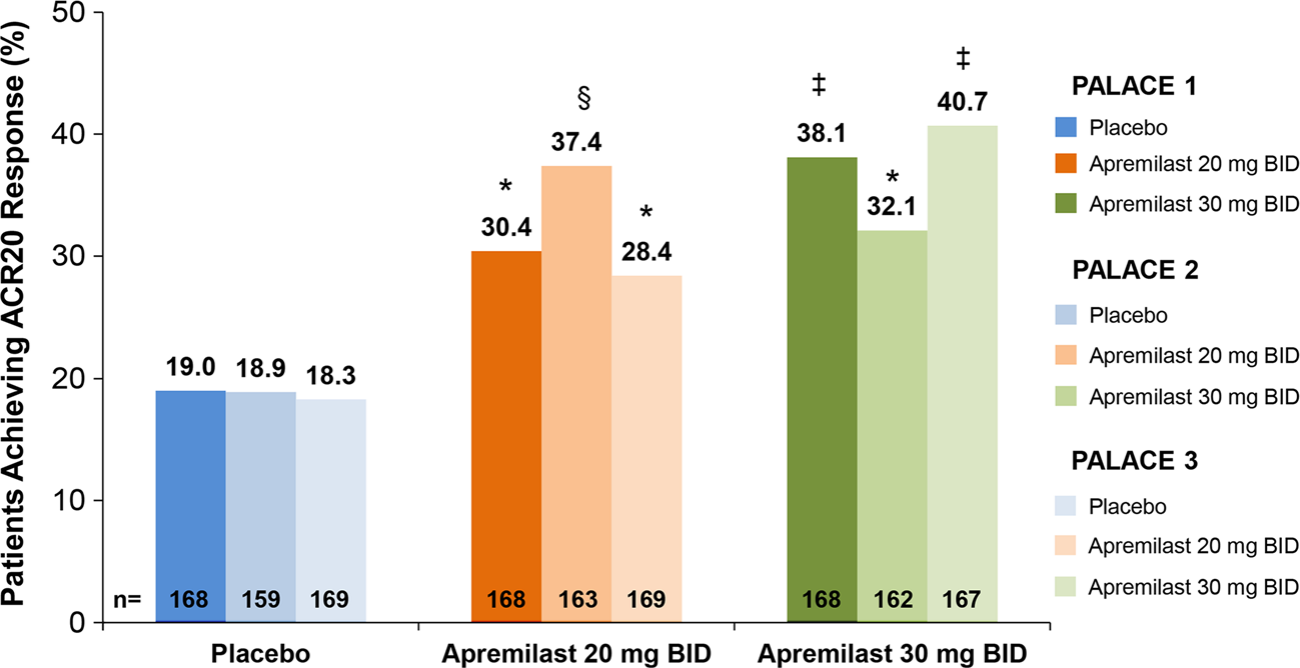
American College of Rheumatology 20 (ACR20) response at week 16 in the Psoriatic Arthritis Long-term Assessment of Clinical Efficacy (PALACE) 1, 2, and 3 trials. The intent-to-treat population includes all randomized patients who received ≥1 dose of study medication. Non-responder imputation was used to handle missing values. *p < 0.05; ^§^ *p* < 0.005; ^ǂ^ *p* ≤ 0.0001 versus placebo. *BID* twice daily. Reproduced with permission from Blanco et al. [33]

In PALACE 1, at Week 16 in the ITT population, 20% improvement from baseline in modified American College of Rheumatology response criteria (ACR20) [32]—the primary end point—was achieved by significantly greater proportions of patients receiving apremilast 20 mg BID [51/168 (30.4%); *p* = 0.0166] and apremilast 30 mg BID [64/168 (38.1%); *p* = 0.0001] compared with patients receiving placebo [32/168 (19.0%)] (Fig. 5) [30]. Similar rates of ACR20 response at Week 16 were observed in PALACE 2 and 3 (Fig. 5) [33]. Analysis of data from PALACE 1, 2, and 3 also showed beneficial effects of apremilast at Week 16 on key symptoms of PsA, including swollen and tender joint counts. Across the three studies, median percent changes in swollen and tender joint counts with apremilast 20 mg BID treatment ranged from −34.9 to 50.0% and −24.2 to 36.2%, respectively. With apremilast 30 mg BID treatment, median percent changes in swollen and tender joint counts ranged from −50.0 to −53.9% and −33.3 to −43.2%, respectively (Table 2) [31].

**Table 2:** Change in swollen and tender joint counts at week 16 and at week 52 (PALACE 1, 2, and 3) [31]

	Placebo			Apremilast					
				20 mg BID			30 mg BID		
	PALACE 1	PALACE 2	PALACE 3	PALACE 1	PALACE 2	PALACE 3	PALACE 1	PALACE 2	PALACE 3
Week 16 (intent to treat, LOCF)	*n* = 166	*n* = 154	*n* = 165	*n* = 164	*n* = 158	*n* = 164	*n* = 164	*n* = 155	*n* = 161
Swollen joint count (0–76), median percent change	−16.7	−33.3	−20.0	−39.1*	−50.0^§^	−34.9*	−50.0^‡^	−53.9^§^	−50.0^§^
Tender joint count (0–78), median percent change	−9.0	−8.7	−7.7	−24.2^§^	−36.2^ǂ^	−29.3^ǂ^	−43.2^ǂ^	−33.3^§^	−42.9^ǂ^

Week 52 (data as observed)	–	–	–	*n* = 121	*n* = 125	*n* = 122	*n* = 131	*n* = 118	*n* = 127
Swollen joint count (0–76), median percent change	**–**	**–**	**–**	−78.8	−87.5	−80.0	−77.8	−87.5	−79.2
Tender joint count (0–78), median percent change	**–**	**–**	**–**	−69.2	−58.3	−66.7	−62.5	−63.1	−70.0

Week 52 data are as observed among patients initially randomized to apremilast 20 mg BID and apremilast 30 mg BID. *p < 0.05; ^§^ *p* < 0.005; ^‡^ *p* ≤ 0.0001 versus placebo based on an analysis of covariance model using the rank transformation

*BID* twice daily, *LOCF* last observation carried forward, *PALACE* Psoriatic Arthritis Long-term Assessment of Clinical Efficacy

**Fig. 6:**
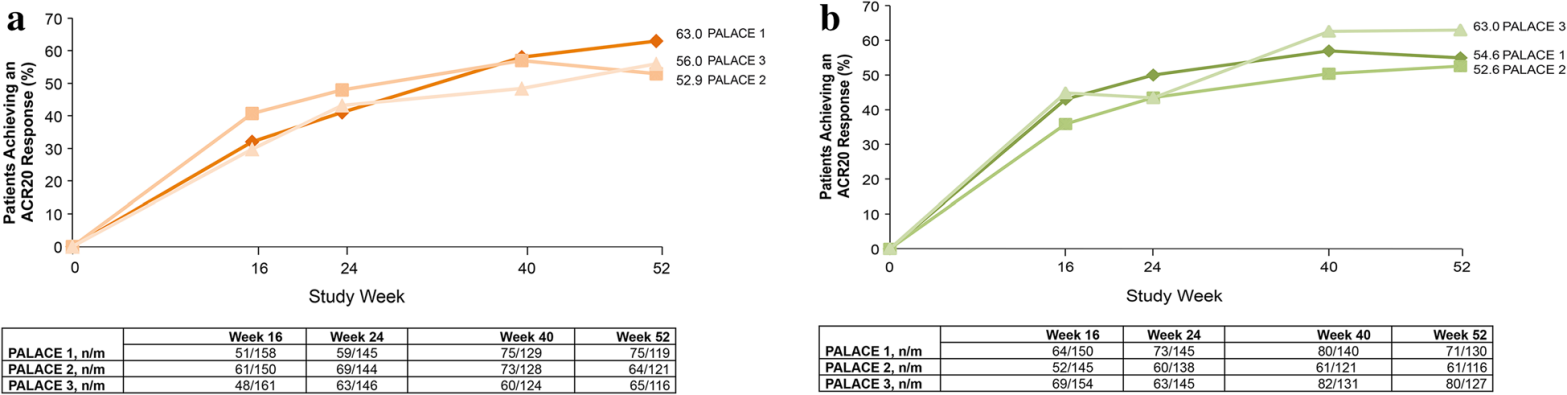
American College of Rheumatology 20 (ACR20) response over 52 weeks in **a** patients initially randomized to apremilast 20 mg twice daily (BID) and **b** patients initially randomized to apremilast 30 mg BID (data as observed). *PALACE* Psoriatic Arthritis Long-term Assessment of Clinical Efficacy. Reproduced with permission from Blanco et al. [33]

The response rates observed in the signs and symptoms of PsA were maintained among patients initially randomized to apremilast at baseline and treated continually over 52 weeks [33]. ACR20 response rates were generally sustained over 52 weeks among patients initially randomized to apremilast in the three pivotal trials (Fig. 6) [33]. At Week 52, the proportions of these patients who achieved ACR20 response ranged from 52.9 to 63.0% with apremilast 20 mg BID and from 52.6 to 63.0% with apremilast 30 mg BID [33]. Improvements in swollen and tender joint counts were also sustained over 52 weeks in these patients. Median percent changes in swollen and tender joint counts with apremilast treatment 20 mg BID ranged from −78.8 to −87.5% and from −58.3 to −69.2%, respectively, across the 3 PALACE trials. Median percent changes in swollen and tender joint counts with apremilast 30 mg BID treatment ranged from −77.8 to −87.5% and −62.5 to −70.0% across PALACE 1, 2, and 3 (Table 2) [31].

### Patient-Reported Outcomes in Psoriatic Arthritis: Impact of Apremilast on HRQOL, Including Physical Function

**Fig. 7:**
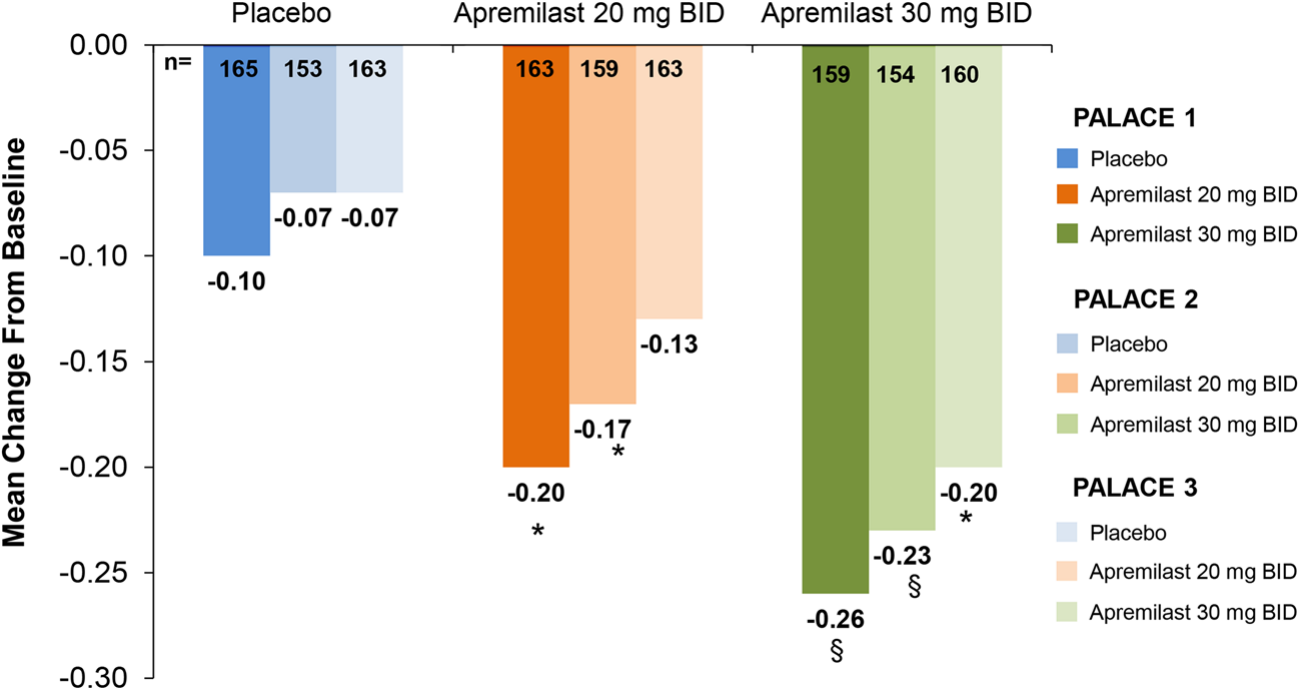
Mean change from baseline in Health Assessment Questionnaire-Disability Index scores at week 16. The intent-to-treat population includes all randomized patients who received ≥1 dose of study medication. *BID* twice daily, *PALACE* Psoriatic Arthritis Long-term Assessment of Clinical Efficacy, *p < 0.05; ^§^ *p* < 0.005 versus placebo, based on the analysis of covariance model. Reproduced with permission from Bird et al. [34]

Apremilast improves HRQOL, including physical function, as observed in the PALACE trials. In PALACE 1, patients receiving apremilast exhibited significantly greater improvements in physical function at Week 16 compared with patients receiving placebo, based on mean decreases from baseline in Health Assessment Questionnaire-Disability index (HAQ-DI) scores (placebo: −0.10; apremilast 20 mg BID: −0.20, *p* < 0.05; apremilast 30 mg BID: −0.26, *p* < 0.005) (Fig. 7) [34]. Similar improvements in HAQ-DI scores were seen at Week 16 in PALACE 2 and 3 [34]. In PALACE 1, 2, and 3, improvements in HAQ-DI scores were sustained over 52 weeks in patients initially randomized to apremilast at baseline (data not shown) [33].

Sustained improvements in physical function with apremilast were also demonstrated in all three PALACE trials based on changes in subscores on the 36-item Short-Form Health Survey version 2 (SF-36v2). In PALACE 1, 2, and 3, sustained improvements from baseline in the SF-36v2 Physical Functioning (PF) domain and physical component summary (PCS) scores were observed in patients initially randomized to apremilast [35]. At Week 52, 51.6–64.2% of patients achieved SF-36v2 PF minimal clinically important differences (MCID) of ≥2.5 improvement, and 66.1–72.7% of patients achieved SF-36v2 PCS MCID of ≥2.5 improvement (Table 3) [35].

**Table 3:** Proportions of PALACE patients achieving MCID in SF-36v2 physical functioning and physical component summary scores at week 52 (data as observed) [33, 35]

	Apremilast					
	20 mg BID			30 mg BID		
	PALACE 1	PALACE 2	PALACE 3	PALACE 1	PALACE 2	PALACE 3
Week 52 (data as observed)	*n* = 168	*n* = 163	*n* = 169	*n* = 168	*n* = 162	*n* = 167
SF-36v2 (0–100), mean change						
PF	7.0	4.1	5.7	5.7	5.0	5.9
PCS	7.8	5.1	6.3	6.5	6.4	5.9
Patients achieving MCID (%)						
SF-36v2 PF, ≥2.5^a^	64.2	51.6	61.2	60.0	53.9	58.3
SF-36v2 PCS, ≥2.5^a^	71.7	66.1	72.7	66.2	69.6	68.3

The *n* reflects the number of randomized patients who received ≥1 dose of study medication; actual number of patients available for each parameter may vary

*BID* twice daily, *MCID* minimum clinically important difference, *PALACE* Psoriatic Arthritis Long-term Assessment of Clinical Efficacy, *PCS* physical component summary score, *PF* Physical Functioning subscale, *SF*-*36v2* 36-item medical outcomes survey short-from questionnaire, version 2

^a^Prespecified threshold based on the literature at the time of protocol development

### Enthesitis/Dactylitis and Psoriatic Skin Measures

**Fig. 8:**
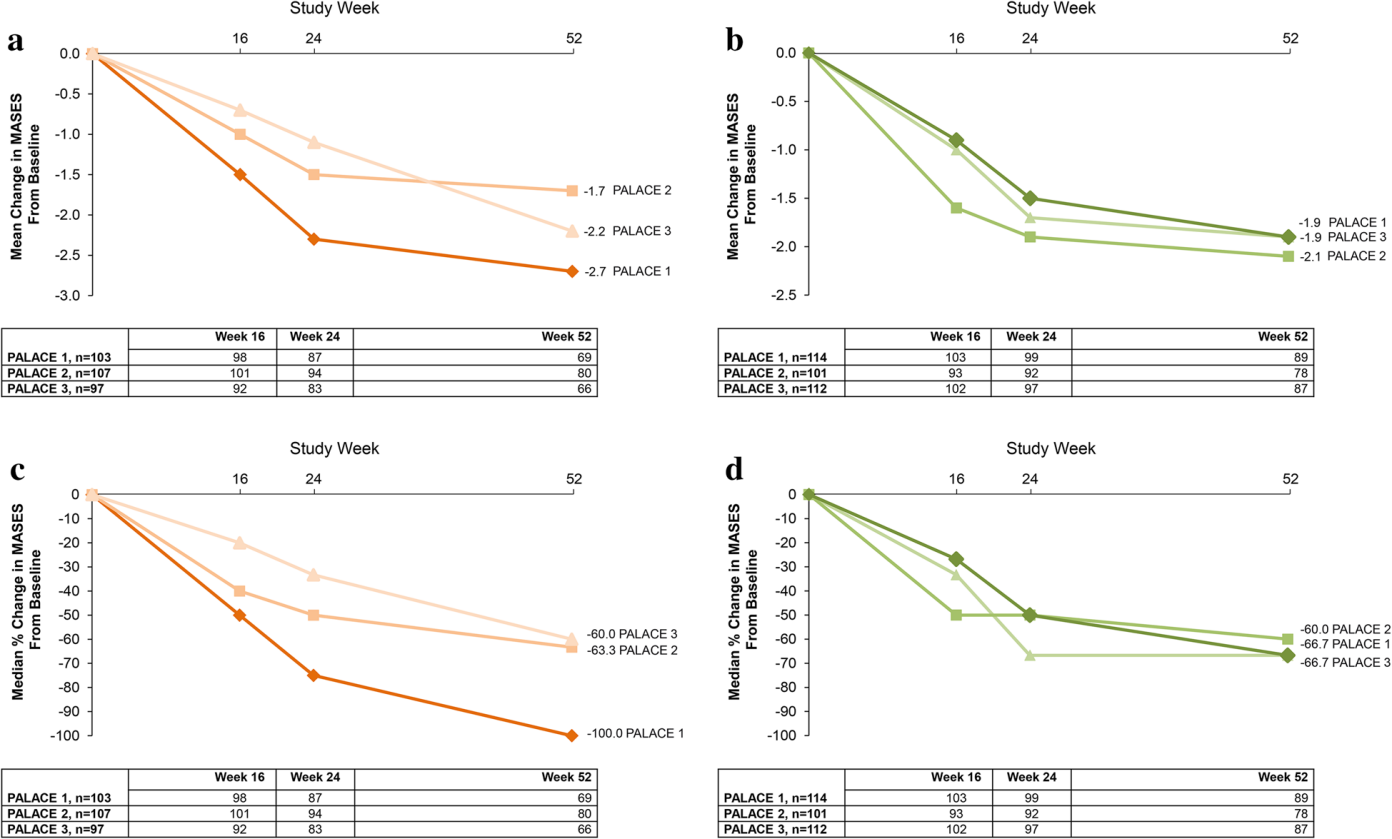
Mean change in Maastricht Ankylosing Spondylitis Enthesitis Score (MASES) over 52 weeks in **a** patients with enthesitis at baseline initially randomized to apremilast 20 mg twice daily (BID) and **b** patients with enthesitis at baseline initially randomized to apremilast 30 mg BID (data as observed); and median percent change in MASES over 52 weeks in **c** patients with enthesitis at baseline initially randomized to apremilast 20 mg BID and **d** patients with enthesitis at baseline initially randomized to apremilast 30 mg BID (data as observed). *PALACE* Psoriatic Arthritis Long-term Assessment of Clinical Efficacy (data on file, Celgene Corporation). Reproduced with permission from Kavanaugh et al. [37]

**Fig. 9:**
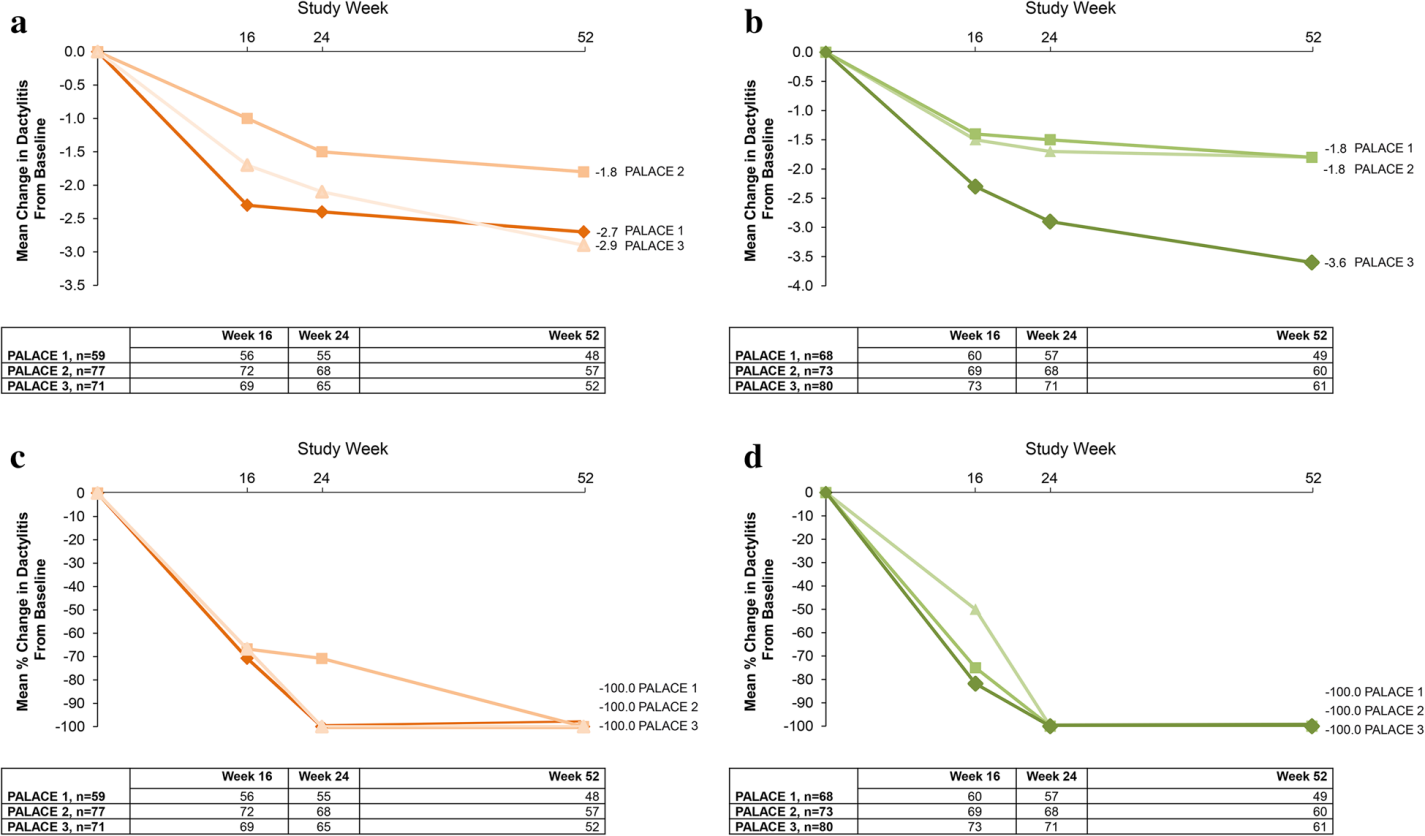
Mean change in dactylitis over 52 weeks in **a** patients with dactilytis at baseline initially randomized to apremilast 20 mg twice daily (BID) and **b** patients with dactylitis at baseline initially randomized to apremilast 30 mg BID (data as observed), and median percent change in dactylitis over 52 weeks in **c** patients with dactylitis at baseline initially randomized to apremilast 20 mg BID and **d** patients with dactylitis at baseline initially randomized to apremilast 30 mg BID (data as observed). *PALACE* Psoriatic Arthritis Long-term Assessment of Clinical Efficacy (data on file, Celgene Corporation). Reproduced with permission from Kavanaugh et al. [37]

Across PALACE 1, 2, and 3, enthesitis was present in 62.5–64.5% of patients at baseline and dactylitis was present in 38.7–44.6% of patients at baseline [36]. In patients with enthesitis and dactylitis at baseline, improvements in Maastricht Ankylosing Enthesitis Score (MASES) and dactylitis count at Week 16 were observed across all three studies [36]. Improvements in enthesitis and dactylitis, as shown by mean changes and median percent changes in MASES and dactylitis count, were sustained over 52 weeks (Figs. 8, 9) (data on file, Celgene Corporation) [37]. At Week 52, the percentage of patients who reached a MASES score of zero across the individual PALACE 1, 2, and 3 studies ranged from 33.3 to 50.7% with apremilast 20 mg BID and 36.8 to 38.2% with apremilast 30 mg BID [36]. Similarly, the percentage of patients achieving a dactylitis score of zero at Week 52 in PALACE 1, 2, and 3 ranged from 57.9 to 75.0% for apremilast 20 mg BID and 63.3–68.9% for apremilast 30 mg BID [36].

**Fig. 10:**
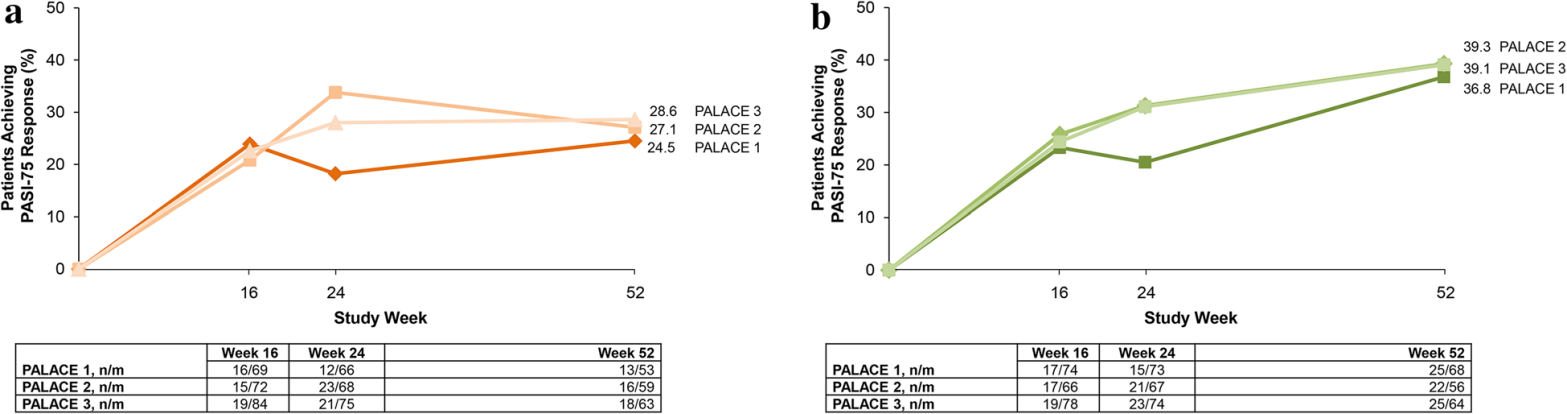
Seventy-five percent reduction from baseline Psoriasis Area and Severity Index (PASI-75) response over 52 weeks in patients with ≥3% body surface area at baseline in **a** patients initially randomized to apremilast 20 mg twice daily (BID) and **b** patients initially randomized to apremilast 30 mg BID (data as observed). *n/m* number of responders/number of patients with sufficient data for evaluation, *PALACE* Psoriatic Arthritis Long-term Assessment of Clinical Efficacy. Reproduced with permission from Kavanaugh et al. [37]

In addition, among patients initially randomized to apremilast at baseline, those with psoriasis body surface area ≥3% at baseline in PALACE 1 experienced sustained improvement in psoriatic skin symptoms, with 24.5% (apremilast 20 mg BID: 13/53) and 36.8% (apremilast 30 mg BID: 25/68) achieving 75% reduction from baseline in Psoriasis Area and Severity Index (PASI-75) score at Week 52 (Fig. 10) [37]; similar sustained PASI-75 response rates at Week 52 were observed in PALACE 2 and 3 [37] (Fig. 10).

Placebo patients who were re-randomized to apremilast at Week 16 or Week 24 demonstrated results in the above efficacy parameters consistent with those seen in patients initially randomized to apremilast (data not shown) [31, 33, 38]. The ongoing open-label phases of the PALACE trials (Fig. 4) [37] will provide valuable information about the long-term efficacy and safety of apremilast treatment.

## Apremilast Safety in Phase III Clinical Trials

Apremilast has demonstrated an acceptable long-term safety and tolerability profile in the PALACE 1, 2, and 3 pivotal trials. In a pooled analysis of safety from patients in these three trials [39], a total of 1,493 patients received study medication and were included in the safety population (placebo: *n* = 495; apremilast 20 mg BID: *n* = 501; apremilast 30 mg BID: *n* = 497). The most common adverse events (AEs) during the weeks 0 to ≥24 apremilast-exposure period were diarrhea, nausea, headache, upper respiratory tract infection (URTI), and nasopharyngitis (Table 4). The proportion of patients reporting diarrhea, nausea, and headache appeared to increase in a treatment- and dose-dependent manner. URTI and nasopharyngitis were reported more frequently with either dose of apremilast compared with placebo, but a dose-related effect was not observed [39]. The nature and severity of AEs and the exposure-adjusted incidence rates were similar in the apremilast-exposure periods (weeks 0 to ≥24 and weeks 0 to ≥52) [39].

**Table 4:** Overview of apremilast safety from PALACE 1, PALACE 2, and PALACE 3 (pooled analysis) [39]

	Placebo-controlled phase (weeks 0 to 24)^a^		Apremilast-exposure period (weeks 0 to ≥ 24)^b^				Apremilast-exposure period (weeks 0 to ≥ 52)^b^			
			Apremilast				Apremilast			
	Placebo *n* = 495		20 mg BID *n* = 720		30 mg BID *n* = 721		20 mg BID *n* = 720		30 mg BID *n* = 721	
Patients	*n* (%)	EAIR/100 patient-years	*n* (%)	EAIR/100 patient-years	*n* (%)	EAIR/100 patient-years	*n* (%)	EAIR/100 patient-years	*n* (%)	EAIR/100 patient-years
AE summary										
≥1 AE	235 (47.5)	200.7	456 (63.3)	201.2	476 (66.0)	218.6	524 (72.8)	163.5	534 (74.1)	174.0
≥1 serious AE	19 (3.8)	11.5	34 (4.7)	7.2	35 (4.9)	7.5	49 (6.8)	6.5	52 (7.2)	7.0
≥1 severe AE	19 (3.8)	11.5	38 (5.3)	8.2	47 (6.5)	10.3	48 (6.7)	6.5	60 (8.3)	8.2
AE leading to drug withdrawal	21 (4.2)	12.5	48 (6.7)	10.1	51 (7.1)	10.8	54 (7.5)	7.1	60 (8.3)	7.9
Death	0 (0.0)	0.0	1^c^ (0.1)	0.2	0 (0.0)	0.0	1^c^ (0.1)	0.1	0 (0.0)	0.0
Most common AEs in any treatment group										
Diarrhea	14 (2.8)	8.5	80 (11.1)	18.7	109 (15.1)	26.2	88 (12.2)	12.8	118 (16.4)	17.7
Nausea	23 (4.6)	14.0	63 (8.8)	14.2	94 (13.0)	21.9	71 (9.9)	9.9	111 (15.4)	16.2
Headache	23 (4.6)	14.1	56 (7.8)	12.6	72 (10.0)	16.3	65 (9.0)	9.1	80 (11.1)	11.3
URTI	15 (3.0)	9.1	55 (7.6)	12.3	54 (7.5)	11.9	82 (11.4)	11.6	67 (9.3)	9.3
Nasopharyngitis	13 (2.6)	7.8	41 (5.7)	9.0	35 (4.9)	7.7	57 (7.9)	7.9	50 (6.9)	6.8

*AE* adverse event, *BID* twice daily, *EAIR* exposure-adjusted incidence rates, *URTI* upper respiratory tract infection

^a^Includes the data through Week 16 for placebo patients who escaped and data through Week 24 for all other placebo patients

^b^Includes all patients who received ≥ 1 dose of apremilast, regardless of when apremilast started

^c^Multiorgan failure not suspected by the investigator to be treatment-related

Diarrhea and nausea, the most common gastrointestinal AEs, occurred most often in the first 2 weeks of exposure to apremilast, with a reduced incidence of these AEs after the first month of dosing [39]. Nausea and diarrhea were predominantly mild or moderate in severity in the weeks 0 to ≥24 apremilast-exposure period [40]. During the weeks 0 to ≥52 apremilast-exposure period, no new severe AEs of diarrhea, nausea, URTI, or nasopharyngitis were reported after the weeks 0 to ≥24 apremilast-exposure period [39].

Serious AEs occurred at low rates, were comparable across treatment groups (Table 4) and did not increase with long-term apremilast exposure based on the exposure-adjusted incidence rates [39]. Exposure-adjusted incidence rates for serious infections, including systemic opportunistic infection, were comparable to placebo [39]. One death occurred (apremilast 20 mg BID) due to multiorgan failure, which was considered by the investigator to be unrelated to apremilast treatment [39]. Marked abnormalities in clinical chemistry and hematology laboratory parameters were mostly transient and comparable across treatment groups with no meaningful treatment or dose effect noted during the apremilast-exposure periods (weeks 0 to ≥24 and weeks 0 to ≥52) [39]. Changes in blood pressure and heart rate were not clinically meaningful, and no patient had a change from baseline in Fridericia’s QTc interval >60 ms [39].

Apremilast was associated with weight loss in PALACE 1, 2, and 3. Weight loss is not unexpected, as it has been reported with other PDE4 inhibitors [41]. In these three pivotal trials, weight decrease was reported as an AE in 1.4% of patients treated with apremilast 20 mg BID and 1.8% of patients treated with apremilast 30 mg BID; 0.1% of patients withdrew from the study because of weight decrease [42]. In the weeks 0 to ≥52 apremilast-exposure period, most patients remained within ±5% of baseline weight (apremilast 20 mg BID: 77.0%; apremilast 30 mg BID: 75.8%); overall, 57.3% patients receiving apremilast 20 mg BID and 57.1% receiving apremilast 30 mg BID experienced weight loss [42]. Weight loss did not lead to any overt medical sequelae or manifestations through the apremilast-exposure period and was unrelated to nausea or diarrhea [42].

Investigations in vivo, using a murine model, and in vitro, using human and murine cell line investigations, demonstrate an association between PDE4 inhibition and decreased adiposity. In PDE4 knockout mice, a significantly lower level of white fat was seen, compared with that in wild-type controls; brown fat levels were unaffected [43]. Moreover, PDE4 inhibition has been linked to enhanced cellular cholesterol efflux, by inducing expression of the transporter ABCA1; increases in ABCA1 were also associated with increases in high-density lipoprotein plasma concentrations [44]. Given the weight loss observed in PALACE 1, 2, and 3, body weight should be monitored during apremilast treatment in all patients; unexplained or clinically significant weight loss may warrant discontinuation of apremilast [23].

PDE4 inhibitors such as roflumilast have also been associated with an increase in AEs of depression and suicidal ideation and behavior [41]. In the pooled safety analysis of PALACE 1, 2, and 3, depression or depressed mood was reported in 1.0% (10/998) of patients treated with apremilast compared with 0.8% (4/495) of patients treated with placebo during the 16-week placebo-controlled period [23]. Instances of suicidal ideation and behavior were observed in 0.2% (3/1,441) of patients receiving apremilast compared with no patients receiving placebo (0/495) during the 16-week placebo-controlled period [39]. In a larger pooled analysis from clinical trials of apremilast in patients with PsA, psoriasis or rheumatoid arthritis, two patients receiving placebo committed suicide, whereas no apremilast-treated patients did so [23, 39]. Because depression is frequently comorbid with psoriatic disease [45], it will continue to be challenging to differentiate disease-related versus treatment-related risks of depressive disorders and suicidal ideation/behaviors.

## Practical Points: Apremilast’s Place in Therapy

Currently available algorithms for PsA treatment guide clinicians through treatment choices, beginning with non-steroidal anti-inflammatory drugs, followed by conventional oral DMARDs, followed by biologic agents [46]. Apremilast is a new treatment option that relieves the pain and inflammation of PsA while improving physical function. As a PDE4 inhibitor, it presents a new mechanism of action for the treatment of PsA.

The efficacy and safety profile of apremilast suggests it may be appropriate for use early in the treatment ladder, comparing favorably with conventional oral therapies and biologic agents. Compared with conventional oral therapies, apremilast exhibits a favorable safety profile and is more convenient, with no lifestyle modifications, pre-screening or ongoing laboratory monitoring required. Compared with biologic agents, apremilast is available orally, thus there is no need for injection, it may be more convenient to prescribe, and it has no apparent increased risk of serious infection or malignancy in clinical trials.

Given the clinical benefits observed in patients with PsA, apremilast has been evaluated in psoriasis and based on this, has been approved by the FDA on September 23, 2014 for the treatment of moderate to severe plaque psoriasis in patients who are candidates for phototherapy or systemic therapy. The Efficacy and Safety Trial Evaluating the Effects of Apremilast in Psoriasis (ESTEEM) study is a phase III clinical trial program comprising two randomized, placebo-controlled studies evaluating the efficacy, safety and tolerability of apremilast in the treatment of patients with moderate to severe plaque psoriasis. Both ESTEEM 1 and 2 demonstrated the efficacy of apremilast 30 mg BID over 16 weeks in patients with moderate to severe plaque psoriasis [47–49]. Response was generally maintained in patients who continued for 52 weeks in the maintenance phases of these trials [48, 50]. The open-label extension studies will provide additional valuable information about the long-term efficacy and safety of apremilast for the treatment of psoriasis.

## Conclusion

Apremilast is a PDE4 inhibitor for the treatment of active PsA in adults. In the phase III PALACE trials, apremilast demonstrated efficacy in the signs and symptoms of PsA as well as improvements in physical function and HRQOL, which were generally sustained over 52 weeks of treatment. Ongoing PALACE open-label extension trials of up to 4 years in duration will provide valuable information regarding the long-term clinical effects and safety of apremilast therapy in PsA; the ESTEEM trials are investigating apremilast for the treatment of psoriasis. The safety and tolerability profile of apremilast indicates no need for laboratory monitoring. With an alternative mechanism of action, oral route of administration and comparatively favorable safety profile, apremilast presents a new treatment option for PsA that may be appropriate for use early in the treatment ladder.

## Electronic supplementary material

Below is the link to the electronic supplementary material.

Supplementary material 1 (PDF 199 kb)
