# Erratum to: Apremilast: A Phosphodiesterase 4 Inhibitor for the Treatment of Psoriatic Arthritis

**DOI:** 10.1007/s40744-015-0009-8

**Published:** 2015-02-24

**Authors:** Philip J. Mease

**Affiliations:** Swedish Medical Center and University of Washington School of Medicine, 601 Broadway, Suite 600, Seattle, WA 98122 USA

## Erratum to: Rheumatol Ther (2014) 1(1–2):1–20 DOI 10.1007/s40744-014-0005-4

The author of the above-mentioned paper would like to make the following correction to the first paragraph of the Acknowledgments section on page 16.

“The author received editorial support in the preparation of this report from Kristin Carlin and Jennifer Schwinn, Peloton Advantage, LLC, Parsippany, NJ, USA, funded by Celgene Corporation, Summit, NJ, USA. The author, however, directed and is fully responsible for all content and editorial decisions for this manuscript. The named author meets the ICMJE criteria for authorship for this manuscript, takes responsibility for the integrity of the work as a whole, and has given final approval for the version to be published.”

