# Supplementary material for: Apremilast: A Phosphodiesterase 4 Inhibitor for the Treatment of Psoriatic Arthritis
Source: Rheumatol Ther. 2014 Dec 9;1(1):1–20. doi: 10.1007/s40744-014-0005-4 (PMC4883260; doi:10.1007/s40744-014-0005-4)
Supplement: Supplementary file 1 — Supplementary material 1 (PDF 199 kb) [file 40744_2014_5_MOESM1_ESM.pdf]

- Psoriatic arthritis (PsA) is an underdiagnosed spondyloarthritis associated with psoriasis that is progressive and can lead to impaired physical function and quality of life.
- Apremilast is an orally available phosphodiesterase 4 (PDE4) inhibitor recently approved for treatment of PsA.
- The Psoriatic Arthritis Long-term Assessment of Clinical Efficacy (PALACE) phase III pivotal trials demonstrated that apremilast significantly improves clinical signs and symptoms of PsA, as well as patient-reported physical function and quality of life.
- Apremilast exhibited acceptable safety during up to 52 weeks of treatment in the PALACE pivotal trials, with diarrhea and nausea being the most common adverse events.
- Apremilast presents a new mechanism of action for the treatment of PsA; its efficacy and safety profile indicate that it may be appropriate for use early in treatment.

This summary slide represents the opinions of the authors. Sponsorship for this study was funded by Celgene Corporation, Warren, NJ, USA. Medical writing assistance for this study was provided by Kristin Carlin, Peloton Advantage, LLC, and Jennifer Schwinn. For a full list of acknowledgments and conflicts of interest for all authors of this article, please see the full text online. Copyright © The Authors 2014. Creative Commons Attribution Noncommercial License (CC BY-NC).
